# Weighing and modelling factors influencing serum cortisol and melatonin concentration among workers that are exposed to various sound pressure levels using neural network algorithm: An empirical study

**DOI:** 10.1016/j.heliyon.2020.e05044

**Published:** 2020-09-28

**Authors:** Sajad Zare, Rasoul Hemmatjo, Hossein ElahiShirvan, Ashkan Jafari Malekabad, Reza Kazemi, Farshad Nadri

**Affiliations:** a Department of Occupational Health Engineering, Faculty of Health, Kerman University of Medical Sciences, Kerman, Iran; b Department of Occupational Health Engineering, Faculty of Health, Urmia University of Medical Sciences, Urmia, Iran; c Students' Research Committee, Kerman University of Medical Sciences, Kerman, Iran; d Occupational Health Engineering, Students' Research Committee, Kerman University of Medical Sciences, Kerman, Iran; e Department of Ergonomics, School of Health, Shiraz University of Medical Sciences, Shiraz, Iran; f Department of Occupational Health Engineering, Faculty of Health, Kermanshah University of Medical Sciences, Kermanshah, Iran

**Keywords:** Industrial engineering, Public health, Software engineering, Theoretical computer science, Acoustical engineering, Safety engineering, Noise, Modelling, Cortisol, Melatonin, Neural network

## Abstract

**Background:**

Noise is one of the most common harmful agents in the workplace. Exposure to excessive noise can lead to complications such as cardiovascular disorders, disturbance of body hormones’ rhythm and hearing loss. This study aimed at weighing and modelling factors influencing serum cortisol and melatonin concentrations of workers that are exposed to various sound pressure levels using neural network algorithm.

**Methodology:**

A case-control design was adopted in the current research. The required data were collected from 75 industrial and mining firm staff members. They were assigned to three groups with equal sample sizes (25 workers). In developing the conceptual model in regard to variables that may affect workers’ serum cortisol and melatonin concentration, SPL, age, weight, and height were included. The influence of SPL on serum cortisol concentration as assessed in the three shifts. Moreover, radioimmunoassay (RIA) was utilized to assess serum cortisol and melatonin concentrations. Neural network algorithm was subsequently exploited to weigh and model predictor factors. IBM SPSS Modeler 18.0 was the software program used for data analysis.

**Results:**

The average cortisol concentration values for administrative, condensing, and pelletizing units respectively were 10.24 ± 2.35, 12.15 ± 3.46, and 14.91 ± 4.16$\frac{\mu g}{dl}$. On the other hand, the average melatonin concentration values for administrative, condensing, and pelletizing units respectively were 37 ± 12.52, 34 ± 13.15, and 27 ± 9.54$\frac{\mu g}{dl}$. According to the results of the developed model for cortisol, SPL3 (32%) and age (5%) respectively had the highest and lowest impact. On the other hand, considering the model developed for melatonin, height (27%) and SPL1 (10%) were the most and least influential factors in that order. The accuracy rates of the model were also found to be 95% for cortisol and 97% for melatonin.

**Conclusion:**

Comparing cortisol concentrations during various shifts revealed a significant reduction (from the beginning to the end of the shift) in all the three groups. Further, the rise of SPL would result in higher secretion of cortisol. Moreover, in all the three groups, the average serum melatonin concentration went up from the beginning to the middle of the shift and then declined to the end of the shift. Considering the accuracy rates of the models developed to predict hormones, neural network algorithm is a suitable and powerful tool for weighing and modelling factors influencing serum cortisol and melatonin concentrations.

## 1 Introduction

As an offshoot of the growing rate of industrialization across the world, noise exposure has turned into a typical phenomenon in industrial contexts (Zare et al., 2018). In fact, occupational health is nowadays blamed as a widespread harmful agent and a critical risk factor among industrial workers. Employees in a variety of settings, such as food, fabric, drug, printed material, and metal product manufacturing firms as well as mining, forestry, and construction operations, are commonly exposed to high noise levels (Thepaksorn et al., 2018). Noise exposure is believed to undergo an even faster growth as a result of economic development and urbanization (Lin et al., 2018).

The number of people who are in contact with excessive noise levels (>85 dBA) worldwide is estimated to be over 60 million. Additionally, around 22 million American workers are annually exposed to superfluous noise in their working milieu (Safari Variani et al., 2018; Zare et al., 2019). On the other hand, following conservative estimations, more than one million healthy people began to develop some form of disease each year as a result of exposure to traffic noise in Western Europe (Miedema et al., 2011). Moreover, the results of a study carried out among 30,000 lumber mill Canadian workers indicated a positive association between employment duration and noise level, on the one hand, and increased risk of fata acute myocardial infraction, on the other hand (Davies et al., 2005).

Exposure to excessive noise is a worldwide occupational health issue with various social and physiological repercussions like anatomical, nonauditory and auditory consequences (Nassiri et al., 2017, 2016). Exposure to high noise levels may lead to noise-induced hearing loss (NIHL), an auditory syndrome that causes changes in hearing threshold and deterioration of speech perception. Excessive contact with high noise levels can further have nonauditory impacts on workers’ health. The nonauditory consequences may damage the autonomic nervous system, lead to heightened skin temperature and pulse rate, increase blood pressure, and cause abnormal secretion of hormones, constriction of blood vessels, and muscle tenseness (Fouladi et al., 2012; Nassiri et al., 2016).

According to the latest research findings, circadian rhythm of cortisol – a health biomarker that has been extensively investigated – is controlled by a SCN-PVN (suprachiasmatic nucleus-periventricular nucleus) and a SCN-adrenocortical pathway. Due to the daily rhythm of cortisol, researchers have found it difficult to run an assessment of cortisol levels or diurnal rhythms. Cortisol circadian rhythm is believed to grow within the first hour after waking up and reach its pinnacle around 30–45 min after wake-up. It will then gradually dwindle during the day and will fall to its minimum at midnight. Subsequently, it begins to rise again over nocturnal sleep until the time the individual wakes up during the next morning. The connection between cortisol and various diseases among people from different age groups has attracted a lot of research attention over the past years. Research findings have demonstrated that both physical and mental health are negatively influenced by dysregulation in the secretion or diurnal rhythm of cortisol. Patients suffering from Alzheimer have also been diagnosed with cortisol dysregulation (Barca et al., 2019; Lai et al., 2018).

At night, the synthesis of melatonin is induced by a neural output signal, which is produced by the suprachiasmatic nucleus (SCN), followed by its release into the third ventricle and blood circulation (Zisapel, 2018). Melatonin is generated by the pineal gland and other tissues and enjoys a 24 h oscillation, hence a well-conserved circadian rhythm. The level of melatonin goes up at night (80–120 pg/mL) and measurably drops over the day (2–20 pg/mL). A body of research findings have indicated that there is a direct association between declined melatonin levels and development of various cancers like breast, prostate, colon, lymphoma, liver, etc (de Almeida Chuffa et al., 2019).

Data mining (DM), which is an interdisciplinary subfield of computer science, entails a process pattern extraction from large data through methods that originate from artificial intelligence, machine learning, statistics, and database systems. In other words, DM aims to collect necessary information and transform it to an understandable structure for further use. Accordingly, the extracted data may be classified to predict outcomes after an intervention or the pattern construction may entail studying the association, group, or detection of variable deviation (Zare et al., 2019). Different steps should be taken in data mining: (1) selection of focal variables. (2) Data processing. (3) Exploitation of a range of data mining techniques. (4) Information extraction, and (5) data interpretation. Different DM techniques are used for consecutive classification, prediction, association, clustering, and analysis (Han et al., 2011).

Neural networks constitute tools that are employed to analyze and stimulate nonlinear and uncertain systems, i.e. systems in which the associations between the components and parameters are not easily deciphered (Kohzadi et al., 1995). In the brain, neural networks are trained through processing experimental data. That is, they learn the rules by carrying out calculations on numeral data and examples. Therefore, neural networks are regarded as smart systems. A benefit of neural networks is that they can directly learn from the data without any requirement to estimate their statistical characteristics. Neural networks have the capability of detecting an association between a series of inputs and outputs to run estimations of other outputs without considering any type of primary assumption or prior knowledge about the connection between the examined parameters (Golabi et al., 2013).

It is highly critical to protect the workforce's health and prevent disease or profession-related accidents. Moreover, few studies have focused on the changes in cortisol and melatonin level by examining the weight of various factors. Thus, the current research aimed to:

1. Determine the concentration of serum cortisol and melatonin (target factors) in the three exposure groups in three various times during the night shift.
2. Run a comparison of the average serum cortisol and melatonin concentration of the three exposure groups in three different times during the night shift.
3. Identify the predictor factors for the purpose of weighing and modelling factors that influence serum cortisol and melatonin concentration among workers.
4. Weigh and model factors that influence serum cortisol and melatonin concentration among workers.

## 2 Methods

### 2.1 Study design

Data collection was carried out in 2019 in a mining and industrial firm in southeast Iran. The participants entailed 75 male workers, all of whom completed a written consent form before taking part in the research. Furthermore, their medical records were examined to ensure they did not have any health-related problems (e.g., diabetes, high/low blood pressure, cancer, etc.) prior to the research. The information related to their demographic features was gleaned on the experiment day.

The participants were divided into two case groups and one control group. The three groups were equal in terms of their sample size (N = 25). The case group participants worked in the two factories and were therefore exposed to noise during their working hours. Conversely, the participants in the control group were clerks who took care of office-related duties and were less likely to be in contact with noise. The participants' metabolism in all the three groups was examined following ISO 8996 (ISO, 1990). The factory workers in the case groups attended their workplace following a 3-3-3-3 shift pattern, i.e. 3 mornings, 3 evenings, 3 nights, and 3 days off. With the aim of exploring the impact of work shift on serum cortisol concentration, the participants’ cortisol and melatonin levels were gauged during the night shifts. That is, the researchers were interested in studying fluctuations of serum cortisol concentration caused by various SPLs. Measurements were carried out at the beginning of the shift (11–11:30 PM), 3 h into the shift (2–2:30 PM), and 3 h later (5–5:30 AM). Each measurement was accomplished once only.

In the next stage, the data associated with the predicting variables – including age, weight, height, and SPL) – and the dependent variables (i.e., cortisol and melatonin concentrations) were collected. In the final stage, neural network algorithm was utilized to weigh the variables and generate the model.

### 2.2 Sampling procedure

A control group and two case groups were included in the study. Care was taken to have an equal number of participants in each group. According to prior studies, each group should contain a minimum of 25 individuals to have a power level of 80% and minimize the risk of Type I error (Alpha = 0.05). As a result, 75 workers were assigned to the three groups.

### 2.3 Determining predictor factors

In this study; SPL, age, weight, and height were selected as the predictor factors to develop a model on factors that may influence workers’ serum cortisol and melatonin concentration.

#### 2.3.1 Age, weight, and height

The participants’ age, weight and height were gauged through administering surveys and examining medical records.

#### 2.3.2 Equivalent SPL

Following the ISO 9612 standard, equivalent SPL was measured using dosimetry. Measurement was carried out using a TES-1345 dosimeter (Sunlight Electronic Technology Co. Ltd., China), which was calibrated by a CEL-110/2 calibrator (CASELLA, USA) prior to running assessments (ISO, 2009).

### 2.4 Determining the target factor

In this study, the concentrations of serum cortisol and melatonin were selected as the target factor.

#### 2.4.1 Assessing the concentrations of serum cortisol and melatonin

During the night shift, 5 mL of the participating workers’ blood sample was collected in the three data collection occasions to gauge serum cortisol and melatonin concentration. All the participants were sitting during sampling. The taken samples were transferred to numbered tubes which contained anticoagulant ethylenediaminetetraacetic acid (EDTA). The tubes were then transferred to an authentic medical diagnostic laboratory under controlled condition (i.e. inside ice box). A radioimmunoassay (Diagnostic Products Corporation, Los Angeles, USA) was used to measure serum cortisol concentration (Yalow and Berson, 1996).

### 2.5 Weighing and modelling influential factors using neural network algorithm

Upon measuring blood hormones’ concentrations and collecting data on predictor factors, neural network algorithm was utilized to weigh and model the factors. The factors (predictors & targets) are divided into two parts: Teaching and Testing. In the Teaching part, 20 people were selected from each group, hence the total number of participants was 60. In the Testing part, 5 people were selected from each group, thus having 15 participants in total.

#### 2.5.1 Input and output coding

It is believed to be a positive thing for neural networks to show robustness to unpredictable pattern variations in new data set. On the contrary, the negative aspect of such networks is that they pursue a standardized procedure for encoding attribute values. In other words, a value varying from 0 to 1 is given to all the attributes, including the categorical ones. The calculation algorithm is portrayed in the following equation (Equation 1) (Larose and Larose, 2014).

Eq 1 $$X^{*}=\frac{X-\min\left(X\right)}{range\left(X\right)}=\frac{X-\min\left(X\right)}{\max\left(X\right)-\min\left(X\right)}$$

Single output nodes can be used after vivid ordering of the classes. As an example, reading prowess in elementary school can be categorized based on a group of student-related variables.

The first, second, and third reading levels may respectively range from 0 to 0.25, 0.25 to 0.50, and 0.50 to 0.75. The fourth grade reading level also receives values greater than 0.75 (Larose and Larose, 2014).

#### 2.5.2 Neural networks for estimation and prediction

Given a continuous output is generated in neural networks, according to Eq. (2), they are usually used to carry out estimations and predictions:

Eq. 2 Prediction = output X (data range) + minimum

In this equation, output refers to the neural network output in the (0, 1), data range has to do with the range of original attribute value on the normalized scale, and minimum is associated with the non-normalized scale's smallest attribute value.

Neural networks often consist of a layered, feedforward, completely connected network of artificial neurons, or nodes. The network is constrained to a single flow direction with no looping or cycling because of the nature of feedforward. Each neural network will have a minimum of two layers, with most of them possessing three layers known as input layer, a hidden layer, and an output layer. On the other hand, although most neural networks have one hidden layer (which is sufficient for most purposes), some neural networks may have more than one hidden layer. Additionally, neural networks are completely connected in the sense that all the nodes in a specific level are in association with all the nodes in the next layer (instead of the nodes in the same layer). A weight index (e.g. W1A) is used to describe the connection between each two nodes. The weights are granted values ranging from 0 to 1 at the beginning.

**Figure 1:**
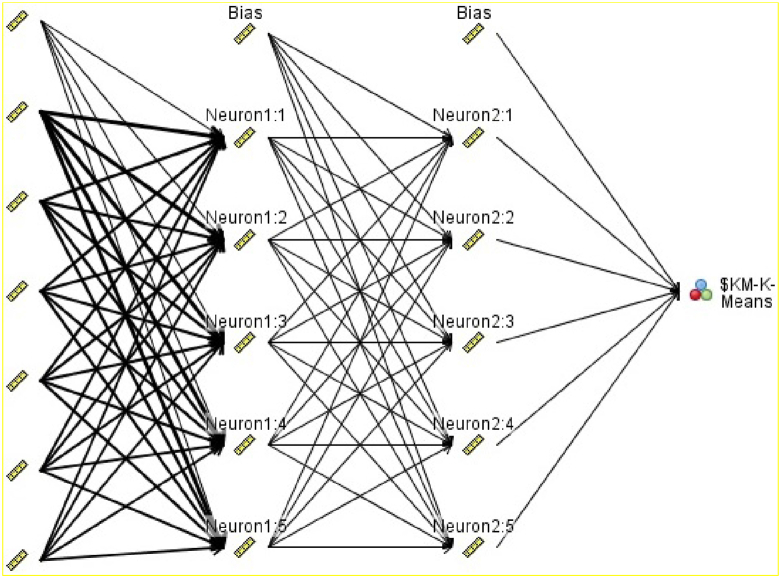
Neural network structure.

The number of input nodes is determined by attributes’ number and type in the data set. Moreover, the output node may have more than one node. This number is a function of the particular undertaken classification task (McCullagh, 2010). Figure 1 illustrates the algorithm function for modeling the hormones.

The number of hidden layer nodes determines the power and flexibility of the network. A large number of hidden layers leads to overfitting, a phenomenon that causes training set memorization undermining generalization to the validation set. Overfitting further reduces hidden layers in number. The number of hidden layer nodes goes up if the training accuracy is too small (Larose and Larose, 2014).

Data set inputs, like attribute values, enter the input layer and directly go through the hidden layer without any processing. This indicates that the structure of input layer nodes is different from that of the hidden layer or output layer nodes. A combination function is used to combine the node inputs and the connection weights into a single scalar value. This combination function is regarded as net and is often presented by summation (Σ) (See Equation 3) (Larose and Larose, 2014).

Eq. 3 Net *j* = ∑*i Wi j xi j* = *W*0 *j x*0 *j* + *W*1 *j x*1 *j* + ·· ·+*WI j xI j*

In this equation, *xij* entails the *i*th input to node *j*, *Wij* has to do with the weight associated with the *i*th input to node *j*, and there are *I* + 1 inputs to node *j*. It should be noted that *x*1, *x*2, …, *x*I refer to inputs from upstream nodes, while *x*0 is a constant input with the same qualities of the constant factor in regression models. It conventionally takes the value *x*0 *j* = 1. As a result, every hidden or output layer node j possesses an “extra” input which is equal to a particular weight *W*0 *j x*0 *j* = *W*0 *j*, such as *W*0*B* for node *B*. This function is illustrated in Eq. (4).

Eq. 4 $$net_{A}=\sum_{i}W_{iA}x_{iA}=W_{0A}\left(1\right)+W_{1A}x_{1A}+W_{2A}x_{2A}+W_{3A}x_{3A}$$

In regard to biological neuron functioning, if the connection between two neurons exceed a particular level, neurons are technically fired and signals are transmitted through them. This process is indicative of the fact that there is no linear relationship between the firing response and the input stimulation increment. A nonlinear activation function is used in artificial neural networks to simulate this biological neuron behavior. The most popular activation function is sigmoid (Krogh and Vedelsby, 1995) (See Equation 5).

Eq. 5 $$y=\frac{1}{1+e^{-x}}$$

In this equation, e entails the bas of natural logarithms.

Node computation should be measured before computing net _*Z*_ (Equation 6):

Eq. 6 $$net_{B}=\sum_{i}W_{iB}x_{iB}=W_{0B}\left(1\right)+W_{1B}x_{1B}+W_{2B}x_{2B}+W_{3B}x_{3B}$$

The outputs from nodes *A* and *B* are combined in node *Z* to yield a weighted sum. In particular, the weights associated with the connections between these nodes are utilized to accomplish this process.

Eq. 7 $$net_{Z}=\sum_{i}W_{iZ}x_{iZ}=W_{0Z}\left(1\right)+W_{AZ}x_{AZ}+W_{BZ}x_{BZ}$$

One should note that the inputs *xi* to node *Z* are not data attribute values. Instead, they are outputs from the sigmoid functions from upstream nodes (Marcoulides, 2005) (See Equation 7):

### 2.6 Determining the error rate and algorithm accuracy

In the classification algorithms, which are used to classify discrete output factors, assessment criteria such as accuracy, confusion matrix, sensitivity, feature, etc. can be used. In the current study, accuracy and confusion matrix were used as the two assessment criteria. Confusion matrix is a square matrix whose number of dimensions is equal to the number of output variable classes. In this matrix, the main diameter indicates the correctly predicted percentage. According to Eq. (8), the model accuracy is the ratio of truly predicted cases to all cases (Marcoulides, 2005).

Eq. 8 $$\mathrm{MSE}=\frac{1}{n}\overset{n}{\underset{i=1}{\sum }}{\left(Y_{i}-{\hat{Y}}_{i}\right)}^{2}$$

### 2.7 Ethical consideration

Ethical approval was gained from the Ethics Committee of Kerman University of Medical Sciences (ID: IR.KMU.REC.1398.120). Moreover, all participants signed a consent form.

### 2.8 Data analysis and processing

The gathered data were fed into the Statistical Package for Social Sciences (SPSS) version 18 (SPSS, Inc., Chicago, Illinois, USA). Linear regression was used for data analysis. Furthermore, IBM SPSS Modeler 18.0 software was exploited to model the cortisol and melatonin hormones.

## 3 Results

### 3.1 Predictor factors

The mean scores of the participants' age, weight, and height are displayed in Table 1. The participants’ average age in the control and the two case groups exposed to 80 and 92 dB sound pressure levels respectively were 29.14 ± 2.14, 29.46 ± 2.8, and 30.4 ± 2.93. Their average weight scores also were 81.12 ± 7.25, 81.56 ± 6.49, and 80.72 ± 9.06.

**Table 1:** Predictor factors of the subjects (n = 75).

Factors	Control group exposed to noise level 67 dBA Mean ± SD	Case group exposed to noise level 80 dBA Mean ± SD	Case group exposed to noise level 92 dBA Mean ± SD
Age (years)	29.14 ± 2.14	29.46 ± 2.80	30.40 ± 2.93
Weight (kg)	81.12 ± 7.25	81.56 ± 6.49	80.72 ± 9.06
Height (cm)	179.56 ± 8.11	179.72 ± 8.03	176.8 ± 6.84

#### 3.1.1 The results of measuring sound pressure level

After conducting dosimetry and measuring the equivalent sound pressure level, it was detected that the participants in the control group were exposed to a noise equal to 67 ± 3 dB. Also, the participants in the case group were exposed to SPLs of 80 ± 4 and 92 ± 4.

### 3.2 The concentrations of serum cortisol and melatonin (target factors)

#### 3.2.1 Cortisol

Table 2 displays the various concentrations of serum cortisol. The average cortisol concentration values for administrative, condensing, and pelletizing units respectively were 10.24 ± 2.35, 12.15 ± 3.46, and 14.91 ± 4.16$\frac{\mu g}{dl}$. The lowest cortisol concentration was 7$\frac{\mu g}{dl}$.

**Table 2:** The concentration of serum cortisol (n = 75).

Target factor			Average in light of time and unit (standard deviation)	Overall average of hormones' concentration in light of units
Cortisol $\left(\frac{\mu g}{dl}\right)$	Administrative	11–11:30 PM	12.32 ± 2.32	10.24 ± 2.35
		2–2:30 AM	9.80 ± 1.44	
		5–5:30 AM	8.60 ± 1.41	
	Condensing	11–11:30 PM	14.92 ± 3.2	12.15 ± 3.46
		2–2:30 AM	11.60 ± 3.32	
		5–5:30 AM	9.92 ± 1.47	
	Pelletizing	11–11:30 PM	18.12 ± 3.94	14.91 ± 4.16
		2–2:30 AM	14.20 ± 3.62	
		5–5:30 AM	12.40 ± 2.61	

#### 3.2.2 Melatonin

Table 3 illustrates the various concentrations of serum melatonin. The average melatonin concentration values for administrative, condensing, and pelletizing units respectively were 37 ± 12.52, 34 ± 13.15, and 27 ± 9.54$\frac{\mu g}{dl}$. The lowest cortisol concentration was 13$\frac{\mu g}{dl}$.

**Table 3:** The concentration of serum melatonin (n = 75).

Target factor			Average in light of time and unit (standard deviation)	Overall average of hormones' concentration in light of units
Melatonin $\left(\frac{\mu g}{dl}\right)$	Administrative	11–11:30 PM	24 ± 3.01	37 ± 12.52
		2–2:30 AM	53 ± 2.86	
		5–5:30 AM	32 ± 2.63	
	Condensing	11–11:30 PM	22 ± 2.68	34 ± 13.15
		2–2:30 AM	52 ± 2.57	
		5–5:30 AM	29 ± 3.14	
	Pelletizing	11–11:30 PM	20 ± 2.57	27 ± 9.54
		2–2:30 AM	39 ± 5.19	
		5–5:30 AM	22 ± 4.53	

### 3.3 Modelling factors predicting serum cortisol and melatonin hormones using neural network algorithm

#### 3.3.1 Cortisol

**Figure 2:**
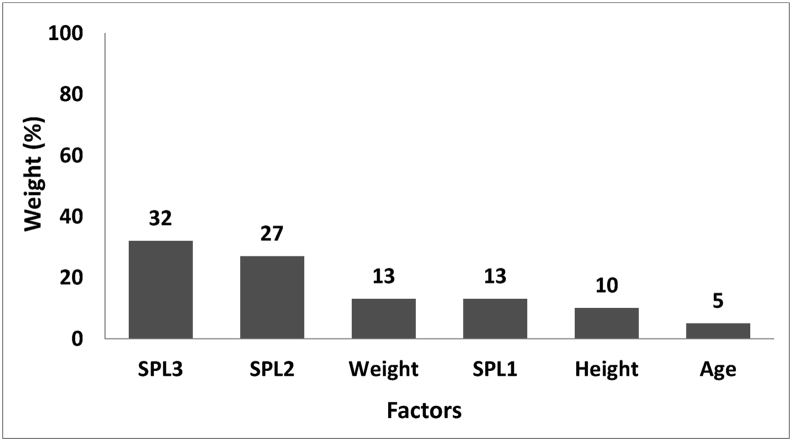
The final model of predictor factors of serum cortisol for workers.

Figure 2 shows the modelling of predictor factors of serum cortisol for the shift workers. As indicated, equivalent sound level (SPL3) of higher than 90 dBA, recorded the highest effect (32%), while age had the lowest influence (5%).

#### 3.3.2 Melatonin

**Figure 3:**
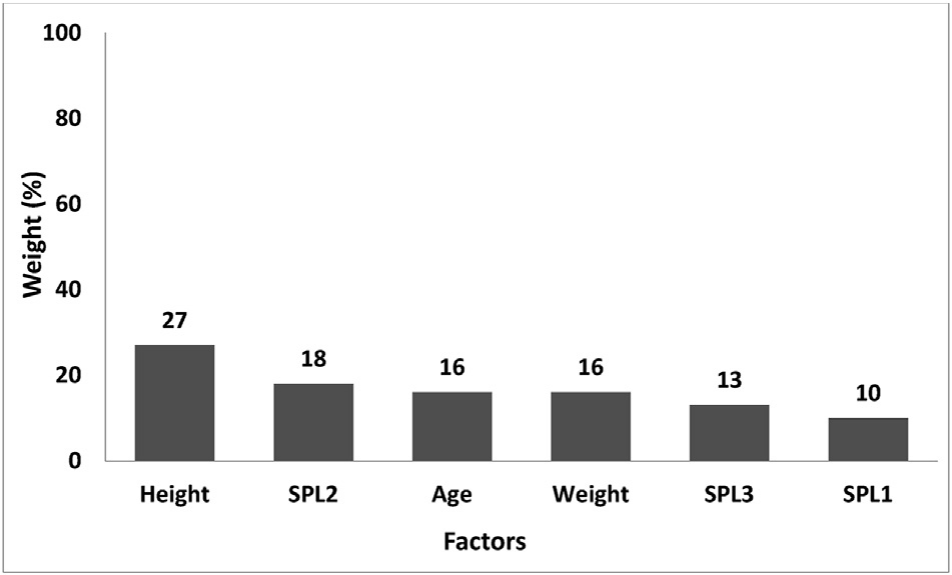
The final model of predictor factors of serum melatonin for workers.

Figure 3 illustrates the modelling of predictor factors of serum melatonin for the shift workers. As observed, height had the biggest effect (27%), followed by SPL2 (18%).

### 3.4 Algorithm's accuracy rate

Table 4 contains information on the algorithm's accuracy and error rate. The accuracy indices of the neural network algorithm for this model were 95% for cortisol and 97% for melatonin.

**Table 4:** The results of accuracy and error rate for models generated by neural network algorithm.

Type of modelling	Accuracy rate	Error rate
Cortisol	95%	5%
Melatonin	97%	3%

## 4 Discussion

This study aimed at weighing and modelling factors that affect the concentration of serum cortisol and melatonin among shift workers in a mining and industrial firm using neural network algorithm. The average sound pressure level to which administrative workers (SPL_1_) were exposed was found to be 77 ± 3 dBA. Furthermore, workers in the condensing (SPL_2_) and pelletizing (SPL_3_) units were respectively exposed to sound pressure levels of 85 ± 3 and 93 ± 4 dBA. Moreover, the average value of the participating workers’ age, weight, and height were 29.67 years, 81.13 kg, and 178.69 cm in that order (Table 1). The average cortisol concentration values for administrative, condensing, and pelletizing units respectively were 10.24 ± 2.35, 12.15 ± 3.46, and 14.91 ± 4.16$\frac{\mu g}{dl}$. On the other hand, the average melatonin concentration values for administrative, condensing, and pelletizing units respectively were 37 ± 12.52, 34 ± 13.15, and 27 ± 9.54 $\frac{\mu g}{dl}$(Tables 2 and 3).

No significant discrepancy was detected between the three groups with respect to their age and body mass index (p > 0.05). Additionally, age and body mass index did not have any statistically measurable impact on cortisol concentration (F = 0.84, p = 0.360, F = 0.23, p = 0.62). On the other side, comparing cortisol concentrations during various shifts revealed a significant reduction (from the beginning to the end of the shift) in all the three groups. It was also found that exposure to higher SPL would lead to cortisol concentration rise; thus, the cortisol concentration was considerably higher in the 92 dBA SPL case group in comparison with the 80 dBA SPL case group and the 67 dBA SPL control group. Besides, SPL3, SPL2, and weight/SPL1 had the highest influence on cortisol secretion in that order with estimated weights of 32%, 27%, and 13% respectively (Figure 2). On the other hand, height (27%), SPL2 (18%), and age (16%) respectively had the highest impact on melatonin secretion (Figure 3). The accuracy rates of cortisol and melatonin modelling were found to be 95% and 97% respectively (Table 4).

In their study, Melamed and Bruhis (1996) explored the influence of chronic exposure to industrial noise on urinary cortisol concentration among 35 industrial workers who were exposed to SPLs over 85 dBA and did not utilize any ear protector. The participants’ urinary cortisol concentration was measured in three different occasions during the day shift (6:30 AM, 10:30 AM, and 1:30 PM). The findings showed that serum cortisol concentration was higher at the end of the work shift than that at the beginning (Melamed and Bruhis, 1996). In the current study, the concentration of serum cortisol was gauged in the case and control groups in three different times (11–11:30 PM, 2–2:30 AM, and 5–5:30 AM) during the night shift. The findings revealed a continuous decrease in cortisol concentration during the night shift. In another study, Brandenberger et al. displayed that there was no significant difference between the case group participants (who were in contact with 85–105 dBA SPLs) and the control group ones with respect to cortisol concentration (Brandenberger et al., 1984). Therefore, their findings are in agreement with the results of the current study. Given that blood sampling is believed to be an aggressive method of data collection, some workers did not agree to participate in this study, which may be a limitation of the research.

Chang et al. (2019) intended to analyze hormone receptor status in primary and recurrent breast cancer through data mining pathology reports. They studied the status of hormone receptors in 3806 patients suffering from breast cancer. After developing a model, it was found that the highest accuracy rates belonged to ER (98.8%), PR (98.7%), and Her-2 (98.4%) in that order (Chang et al., 2019). In a similar vein, the accuracy rates of hormone modelling in the current study were found to be high (98% for cortisol and 97% for melatonin). In another study, Cui et al. (2018) aimed to identify human circadian genes based on time course gene expression profiles by utilizing a deep learning method. Discrimination of the aperiodic genes and the two subtypes of periodic genes was accomplished using DNN. To measure DNN performance, four frequently used machine learning methods – namely k-nearest neighbors, logistic regression, naïve Bayes, and support vector machines – were used for making comparison. DNN model achieved 0.92 in the ROC curve for circadian gene classification of DNN and other four machine learning methods. Moreover, logistic regression and SVM recorded a good performance with scores of 0.81. Nonetheless, the prediction accuracy rates of K-NN (0.74) and naïve Bayes (0.73) were not sufficient (Cui et al., 2018). In the present study, the accuracy rate of models were high (close to 100), similar to that of DNN in Cui et al.‘s study.

Alickovic et al. (2019) ran automatic detection of Alzheimer disease based on histogram and random forest. Indeed, they aimed to detect Alzheimer using ML algorithm. They exploited histogram to transform brain images to feature vectors, which contained the relevant “brain” characteristics. These features subsequently served as the inputs in the classification step. Then, the researchers used the ML algorithm in the classification task to detect Alzheimer. The overall accuracy rate in their study was found to be 85.77% (Alickovic et al., 2019). Similar to Alickovic and Subasi's findings, the accuracy rate of the present study was high. Mehrotra et al. (2011) used Bayesian and logistic classifiers and feature vector to screen polycystic ovary syndrome. They concluded that Bayesian classifier was significantly better than the logistic classifier, with the accuracy rate of Bayesian classifier being calculated as 93.93%. The accuracy rate of logistic classifier, on the other hand, was found to be 91.04% (Mehrotra et al., 2011). Similar to Mehrotra et al.‘s research, the error rate of the current study was less than 10%.

Pryanka N et al. (2017) explored devised usage of naïve Bayes and decision tree (two of the most influential data mining techniques) to estimate heart disease. The results showed that decision tree was more accurate (90%) than naïve Bayes (70%) in the three performed test cases (Priyanka and RaviKumar, 2017). Although the accuracy rate of naïve Bayes in their study was low, the accuracy rate of the decision tree algorithm was at the acceptable range. Likewise, the accuracy rates of the developed models in the current study were over 90%. Simi M S. et al. (2017) assessed women infertility by comparing the accuracy of two different classification algorithms – namely J48 and random forest. The results indicated that the accuracy rate of random forest (96.6%) was higher than that of J48 (86.5%) (Simi et al., 2017). Overall, similar to the current study, the accuracy rates of the two algorithms used by Simi M S et al. were high. With respect to the innovations of the study, two important aspects can be highlighted:

• In this study, the factors that affect the concentration of serum cortisol and melatonin were successfully modeled using neural network data mining algorithm. Most of the previous study on the concentration of hormones did not evaluate the weight and impact of influential factors, a phenomenon that was dealt with in the current study.
• The weight of height, weight, age, and SPL were assessed for shift workers in the proposed model. The majority of previous studies on hearing loss only focused on the error rate and model accuracy, failing to highlight the weight and impact of each of these factors.

As a limitation of the study, it was difficult to establish coordination with the industry and encourage some of the workers to cooperate in conducting the serum hormones.

## 5 Conclusion

The study explores the concentration of serum cortisol and melatonin among shift workers, leading to the development of a model by the use of neural network algorithm. It was found that, during the night shift, SPL and exposure time have considerable effects on cortisol concentration. In particular, the increase of SPL results in measurable rise in cortisol concentration. Extension of exposure time, however, reduces cortisol concentration. According to the results of the developed model for cortisol, SPL3 (32%) and age (5%) respectively had the highest and lowest impact. Furthermore, the accuracy rate of the developed model was found to be 95%. On the other hand, considering the model developed for melatonin, height (27%) and SPL1 (10%) were the most and least influential factors in that order. The accuracy rates of the model were also found to 97%.

## Declarations

### Author contribution statement

Sajad Zare: Conceived and designed the experiments; Contributed reagents, materials, analysis tools or data.

Rasoul Hemmatjo & Farshad Nadri: Analyzed and interpreted the data.

Hossein ElahiShirvan: Performed the experiments; Wrote the paper.

Ashkan Jafari Malekabad: Performed the experiments.

Reza Kazemi: Contributed reagents, materials, analysis tools or data.

### Funding statement

This work was supported by the Institute for Futures Studies in Health at 10.13039/501100004621Kerman University of Medical Sciences (code number: 97000962).

### Competing interest statement

The authors declare no conflict of interest.

### Additional information

No additional information is available for this paper.
